# Modern Innovative Solutions in Improving Outcomes in Chronic Obstructive Pulmonary Disease (MISSION COPD): A Comparison of Clinical Outcomes Before and After the MISSION Clinic

**DOI:** 10.2196/resprot.6850

**Published:** 2017-06-05

**Authors:** Eleanor Lanning, Claire Roberts, Ben Green, Thomas Brown, Will Storrar, Thomas Jones, Carole Fogg, Ann Dewey, Jayne Longstaff, Paul Bassett, Anoop J Chauhan

**Affiliations:** ^1^ Portsmouth Hopitals NHS Trust Cosham United Kingdom; ^2^ University of Portsmouth Portsmouth United Kingdom; ^3^ Stats Consultancy Amersham United Kingdom

**Keywords:** COPD, chronic obstructive pulmonary disorder, United Kingdom, patient questionnaires

## Abstract

**Background:**

Chronic obstructive pulmonary disorder (COPD) affects over 1 million people in the United Kingdom, and 1 person dies from COPD every 20 minutes. The cost to people with COPD and the National Health Service is huge – more than 24 million working days lost a year and the annual expenditure on COPD is £810 million and £930 million a year.

**Objective:**

We aim to identify patients with COPD who are at risk of exacerbations and hospital admissions as well as those who have not been formally diagnosed, yet remain at risk.

**Methods:**

This mixed-methods study will use both data and interviews from patients and health care professionals. The project Modern Innovative SolutionS in Improving Outcomes iN COPD (MISSION COPD) will hold multidisciplinary carousel style clinics to rapidly assess the patients’ COPD and related comorbidities, and enhance patient knowledge and skills for self-management.

**Results:**

This study is ongoing.

**Conclusions:**

This research will capture quantitative and qualitative outcomes to accompany a program of quality improvement through delivery of novel care models.

## Introduction

### Epidemiology of COPD and Prevalence Gap

In the United Kingdom, 1 million people have diagnosed chronic obstructive pulmonary disease (COPD), which accounts for 25,000 deaths annually [1], making it the 5th commonest cause of death and 2nd largest cause for hospital admissions. Annual health care expenditure on COPD is £810 million [2] (equivalent to £1.3 million per 100,000 population). Severe, exacerbation prone COPD costs ten times more to treat than mild disease. COPD causes 24 million lost working days annually, more than any other respiratory condition, costing the economy £3.8 billion [2]. Worse still, cases of COPD will increase by more than 30% in the next 10 years [3], while an estimated 2 million individuals presently remain undiagnosed. Hospital admissions due to COPD have increased by 13% since 2008 [3].

Portsmouth has significantly higher than United Kingdom national averages for prevalence of smoking (main risk factor for COPD), COPD admissions, readmissions, and deaths from COPD (eg, 74.2 vs 51.5/100,000 population) [4]. COPD also contributes to the gap in life expectancy in those living in areas of highest and lowest deprivation confirming significant inequalities [5]. There are still many general practices with a “prevalence gap” between expected and actually diagnosed COPD patients.

The cost burden of COPD to the Portsmouth Hospitals Trust and to the community is high. In 2013/2014, there were 946 hospital contacts for COPD at a cost of £2,176,675. This includes 286 admissions for COPD with respiratory failure—this is associated with a longer length of stay and higher costs of ventilatory support in a high-care environment. In Portsmouth City, there are approximately 4000 patients with a known diagnosis of COPD.

The identification of patients with undiagnosed COPD will lead to an increase in costs in the short term including medication, smoking cessation, referrals to pulmonary rehabilitation, and flu vaccination. However, in the longer term, it will lead to health care savings by preventing acute admissions as the first diagnosis of COPD (15% of COPD diagnosis are made during an acute admission [1]) and slowing disease progression. A case-finding exercise in nearby Southampton increased COPD prevalence in the area by 50% but reduced acute admissions significantly [6].

The goal of Modern Innovative SolutionS Improving Outcomes iN COPD (MISSION COPD) is to target patients who are high risk and severe patients who have a higher chance of admission and especially of admission with respiratory failure.

### Mission Copd

MISSION COPD is a quality improvement project funded by the Health Foundation to trial an innovative way of finding and assessing patients with COPD and also case finding new diagnoses of COPD. MISSION COPD will allow swift early specialist multidisciplinary interventions in primary care to diagnose and treat those at greatest risk, consistent with the United Kingdom’s National Health Services (NHS) “5-year Forward View”, calling for removal of traditional primary and secondary care barriers. MISSION COPD will target COPD patients with risk factors for exacerbations and deteriorating lung function as well potential new diagnoses of COPD.

A Cochrane review published in October 2013 identified 25 randomized controlled trials of integrated disease management interventions in COPD [7]. The Cochrane review concluded that integrated disease management improved quality of life and reduced hospital admissions and highlighted the following areas as important: education/self-management, exacerbation action plan, exercise, psychosocial/ occupational, smoking, optimal medication, nutrition, follow-up, case management, and multidisciplinary approaches.

The MISSION clinics provide all of the important factors identified by the Cochrane review.

#### Stage 1: Identifying Those at Risk

The GRASP-COPD tool, recommended by NHS-IQ (NHS-Improving Quality) will be used to identify patients in a standardized format from practice computer systems using pre-identified Read Codes. Read codes are codes used by all general practitioner (GP) medical record systems to code contact with patients. These are standard across all GP software platforms (eg, EMIS, EMIS Web, SystemOne, Vision).

High-risk COPD patients will then be identified by criteria including smoking status, prescribed medications, frequent antibiotics, prescription of oral steroids, hospital or emergency department (ED) admissions, lung function, and symptoms. Potential new diagnoses of COPD will be identified by searching for patients over 35 with a smoking history and contact with the practice for respiratory symptoms such as bronchitis. Many patients will have ≥2 risk factors. A specialist nurse will review the results to check that patients meet the criteria and to exclude any patients who are under hospital follow-up. All hospital letters are stored on the GP medical records. An invitation to attend the MISSION COPD clinics will then be sent by GP practices.

#### Stage 2: Rapid Access COPD Clinics–Rapid Primary Care Clinics

**Figure 1:**
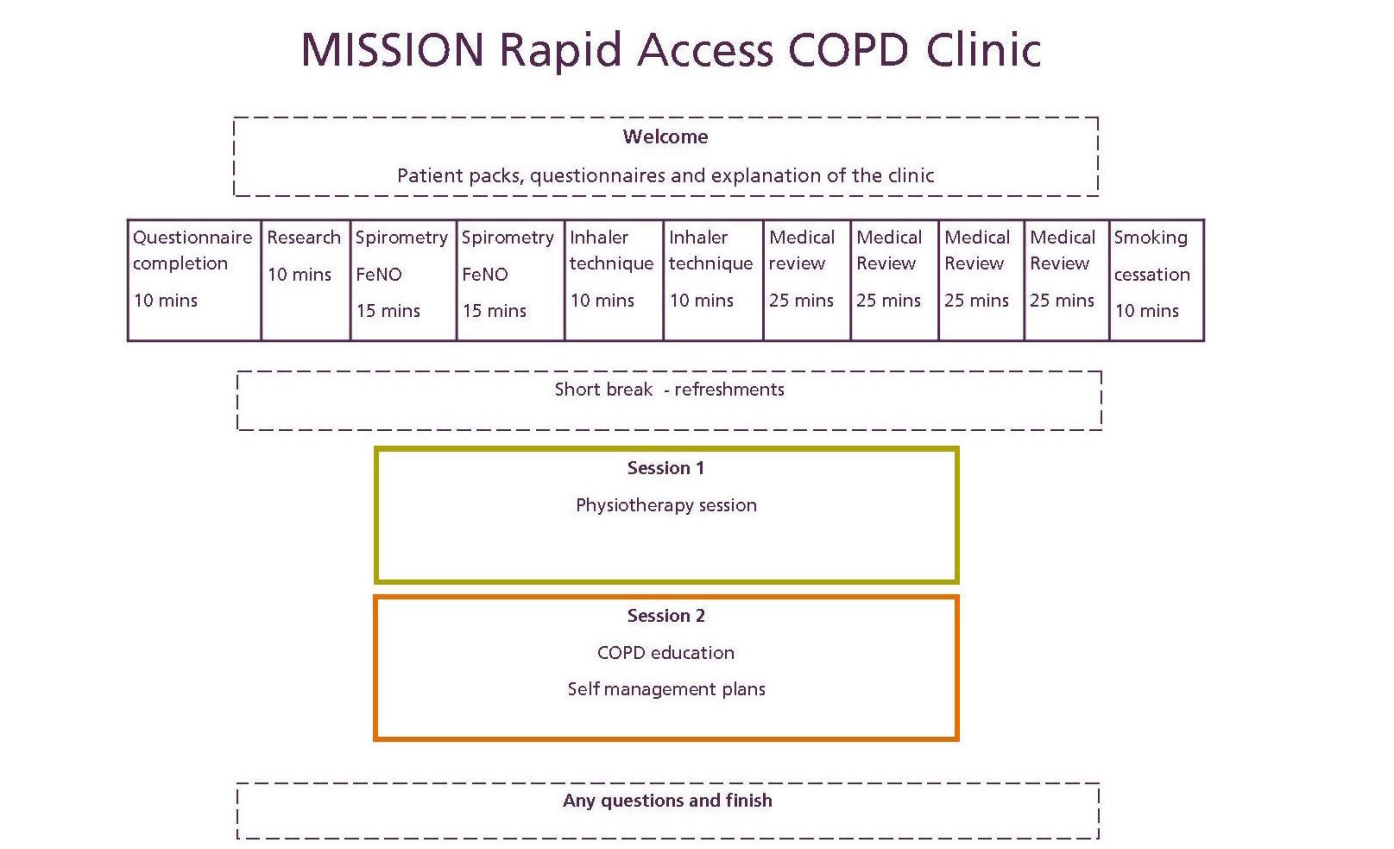
RACC Structure.

MISSION Rapid Access COPD Clinics will provide a comprehensive, multidisciplinary assessment of patients with high-risk COPD and not known to secondary care teams, delivered in a carousel fashion at five GP practices on weekends to overcome barriers in accessing health care such as transport and carer availability (see Figure 1).

At each station, the patients will undergo:

- medical review with comorbidity assessment (eg, gastroesophageal reflux, sleep apnea, anxiety, and depression)
- questionnaire assessments of disease control (COPD Assessment Test [CAT]), quality of life (St George’s Respiratory Questionnaire [SGRQ]), physical activity (Veterans Specific Activity Questionnaire [VSAQ]), and comorbidity, eg, Hospital Anxiety and Depression Score (HADS), gastroesophageal reflux disease questionnaire (GERD-Q), Epworth Sleepiness Score (ESS) for sleep apnea, and Patient Activation Measurement (PAM) for self-management
- spirometry (FEV1, FVC, FEV1/FVC ratio) and exhaled nitric oxide measurement
- inhaler technique assessment and teaching
- smoking cessation advice and referral where needed
- group education: disease process, exacerbations, treatment options, self-management, pulmonary rehabilitation, and breathing control
- receipt of a personalized MISSION COPD Health Plan (copied to GP) encompassing all relevant information and direct referral for pulmonary rehabilitation if necessary
- invitation to participate in clinical research by research team, if eligible to take part in any studies

#### Stage 3: Severe COPD Assessment Clinics‒Comprehensive Secondary Care Clinics

Those with severe disease (according to national guidelines) or multiple comorbidities will be invited for further comprehensive review at MISSION Severe COPD Assessment Clinics, again delivered in carousel-style stations, that may include additional full lung function testing, clinical psychology, dietitian, exercise education, medicines use review, social care advice, and same day computerized tomography (CT) scan of the chest, blood test, and echocardiogram. Both clinics will end with an individualized multidisciplinary team assessment of each patient to plan their future care pathway.

#### Follow-Up

After the MISSION COPD clinics, all patients receive a personalized care plan and the results of the investigations. A copy is also sent to the GP. As part of the service evaluation, a questionnaire will be sent to the patients at 3 and 6 months to measure any change in symptoms or disease control (CAT and SGRQ) as well as self-reported exacerbation history and satisfaction with the clinics. The GP records will be reviewed at 6 months to assess any improvement in exacerbations, hospitals admissions, or GP contact following the clinics.

### MISSION COPD Research Study Overview

MISSION is a new and novel way of delivering highly specialized COPD care and has the potential to change the way COPD care across the United Kingdom is delivered as well as services for other long-term health conditions. The MISSION model is the first model of this type, and the current research study aims to evaluate its success.

The study is a mixed-methods evaluation of the new service comparing outcomes before and after the MISSION clinic using data analysis and self-completion questionnaires.

Retrospective quantitative analysis of the data collected during the MISSION clinics will be analyzed looking for COPD phenotypes and contributing comorbidities. The data collected at the initial visit and 3 and 6-month follow-up will be analyzed looking for improvement in COPD control and quality of life following patient attendance at MISSION clinics.

A self-completion questionnaire will be filled in after the clinic by participants in the research study to assess their views on the clinic and where they would like changes to be made. A questionnaire will also be given to health care professionals to seek their views on the clinic.

Qualitative interviews will be conducted with a sample of willing participants to explore the experiences and acceptability of the participants and health care professionals. The interviews with health care professionals will explore their thoughts on MISSION and its strengths and weaknesses as well as any suggestions for improvement.

### Study Aims and Objectives

#### Aims

Our aim is to use quantitative methods to explore the impact of the MISSION service on clinical outcomes and to retrospectively analyze the data collected (as part of their clinical care) from all patients attending MISSION on COPD control, medication usage and technique, exacerbations, comorbidities, allergies, and investigations (blood tests, radiological imaging, lung function). Additionally, we wish to conduct a prospective qualitative study exploring patients’ and health care professionals’ views on MISSION.

#### Primary Objectives

Our two primary objectives of this study are to assess (1) whether the number of COPD exacerbations (prednisolone or equivalent ≥30 mg for >3 days or antibiotics for >3 days) improves in MISSION patients in the 6 months after the clinic compared with the 6 months before and (2) whether hospital admissions change in the 6 months after the clinic.

#### Secondary Objectives for Patients With Known COPD

For those patients previously diagnosed with COPD, our objectives are to assess the following:

1. whether the number of nonelective GP visits for COPD change in the 6 months after the clinic
2. severity of COPD by Global initiative for Obstructive Lung Disease and British Thoracic Society stage in the MISSION clinics
3. medication and therapy used for COPD in the MISSION clinics and changes made by clinics
4. number of antibiotic courses without prednisolone for lower respiratory tract infections in 6 months before and after MISSION [8]
5. Patient Activation Measure before and after MISSION [8]
6. frequency and severity of comorbidities in the COPD population measured with validated questionnaires (GERD, HADS, ESS) and suspected or confirmed through clinical review
7. COPD control by CAT questionnaire at 3 and 6 months
8. exercise tolerance at 3 and 6 months
9. change in comorbidities at 3 and 6 months
10. inhaler technique – correct technique or correct device
11. lung function and phenotypes of COPD patients seen in MISSION clinic
12. frequency of smoking and referrals for smoking cessation
13. acceptability of MISSION as a service model for patients and staff
14. patient experiences of the MISSION service
15. health economic impact of the MISSION service

#### Secondary Objectives for Patients With Newly Diagnosed COPD

Additionally, we aim to assess (1) the number of patients with newly diagnosed COPD, (2) how long symptoms were present before diagnosis, (3) frequency of smoking and referrals for smoking cessation, (4) antibiotic history, medication history, and number of contacts with respiratory symptoms prior to diagnosis, and (5) lung function and phenotypes.

## Methods

### Design

This is a mixed-methods study. It comprises a quantitative analysis of medical records relating to COPD in patients attending the MISSION clinics for baseline health care usage, and further episodes at 3 and 6 months. Self-completion questionnaires will be used to explore participant and health care professionals’ views on MISSION using closed and open questions. Qualitative telephone interviews and focus group interviews will be used to explore the experiences and acceptability of patients and health care professionals.

### Setting

Data for quantitative analysis will be collected from clinical records made by the clinical team during the MISSION clinics and follow-up questionnaire assessments. GP records will be entered as usual by GP practices. The self-completion questionnaire will be completed by participants in the MISSION research study at the end of the MISSION clinic visit.

Participants will also be invited to give their views on MISSION in a one-to-one interview. The interviews will be held after the clinic by telephone within 2 months of the clinic. Health care professionals will be invited to take part in group interviews at the end of the clinic.

### Participants

Participants will be patients who attend MISSION COPD clinics, identified as having uncontrolled or potentially severe COPD or unrecognized COPD from GP records by the MISSION clinical team or health care professionals who attend the clinic in a clinical capacity. Each patient will act as their own control, with their disease control pre- and post-MISSION clinic providing the comparator data [8].

#### Inclusion Criteria: Patients

The participant must meet all of the following criteria to be considered eligible for the study: male or female, aged 18 years or above; attended the MISSION clinic as a patient; and participant is willing and able to give informed consent for participation in the study. The participant may not enter the study if unable or unwilling to give consent.

#### Inclusion Criteria: Health Care Professionals

The participant must meet all of the following criteria to be considered eligible for the study: male or female, aged 18 years or above; attended the MISSION clinic as a health care professional; and participant is willing and able to give informed consent for participation in the study. The participant may not enter the study if unable or unwilling to give consent.

### Sample Size

All 150 patients who are booked to attend the MISSION COPD clinic will be invited to take part in the study. All health care professionals from primary and secondary care who attend the clinics in a professional capacity will be approached for participation in the self-completion questionnaire and group interview.

The sample size was based on comparing the number of exacerbations in the 6-month period before and after the MISSION intervention. Based on clinical experience, we expect the number of exacerbations in the “pre” period to be approximately 1.0. It is conservatively assumed that the standard deviation of the differences in number of pre- and post-exacerbations will have an equivalent value (ie, SD 1.0).

A clinically important improvement would be obtained post intervention if the number of exacerbations reduced by a third of an exacerbation (ie, on average, one in three patients had one fewer exacerbations). Using a 5% significance level and 90% power, it is calculated that 97 subjects are required for the study.

It is estimated that two-thirds of available patients will agree to participate and complete the study. Therefore, to obtain the calculated number of patients, approximately 150 patients will be approached to participate. This is approximately the same number of patients who are likely to attend the MISSION clinics.

### Recruitment

#### Patients

All patients who are booked to come to the MISSION clinic will receive a MISSION COPD Participant Information Sheet (PIS) with their booking letter. They will be sent this letter at least a week prior to the appointment.

At the end of the MISSION COPD clinic, all patients will be asked if they wish to enroll in the study and will be given the opportunity to ask questions. If they wish to take part, they will be consented by a member of the research team who will be at the MISSION COPD clinic.

#### Health Care Professionals

Health care professionals who attend the MISSION clinics (excluding the research team) will be sent a PIS in advance of the clinic. The initial contact and PIS will be made by a clinical administrator rather than the PI or CI. If they wish to take part, they will be consented by a member of the research team at the MISSION COPD clinic.

#### Screening and Enrollment

##### Quantitative Data

All patients who attend the clinic will be approached for consent having received a PIS prior to the clinic. They will be given the opportunity to discuss the study with a member of the research team and, if they wish, will be able to take the consent form away and send it back in a stamped addressed envelope. If a patient takes the consent form away to fill in at home, they will be contacted by telephone after 2 weeks as a reminder. If at this point they do not wish to take part, they will not be contacted for the study again.

Patients who attend will be asked to fill in follow-up questionnaires at 3 and 6 months by mail. All participants will be asked to complete a self-completion questionnaire on their experience and views of the MISSION clinic (eg, how they feel managing their COPD, do they get enough support from GP/hospital/practice nurse, how they rate the MISSION clinic with free text for comments).

##### Qualitative Data

###### Patients

All patients will be approached for consent having received a PIS prior to the clinic. They will be given the opportunity to discuss the study with a member of the research team and, if they wish, will be able to take the consent form away and send it back in a stamped addressed envelope. If a patient takes the consent form away to fill in at home, they will be contacted after 2 weeks by telephone as a reminder. If at this point they do not wish to take part, they will not be contacted for the study again.

All participants in the study will be invited to take part but may not be selected for interview as we will select on the basis of multiple variation using a grid to map (for example) representation of male/females, age range, job status, and new or existing diagnosis of COPD. We anticipate that there will be up to 30 interviews.

###### Health Care Professionals

All health care professionals will be approached for consent having received a PIS prior to the clinic by email or post. The PIS will be sent by a clinical administrator rather than the Principal Investigator or Chief Investigator. They will be given the opportunity to discuss the study with a member of the research team.

All health care professionals will be asked if they wish to take part in a focus group meeting at the end of the clinic to discuss positives and negatives of the clinic, any improvements, and any suggestion for development in the future. All health care professionals who wish to take part will be included. There will be approximately 11-20 health care professionals at each clinic from both primary and secondary care. An approximate target of 20 health care professionals has been set (three per clinic) with no maximum or minimum number set.

### Ethics

The study will not be initiated before the protocol and all study relevant material such as informed consent forms and participant and GP information sheets have received approval from the Research Ethics Committee (REC), and the respective NHS Research & Development (R&D) department. Any changes to protocol or relevant study documents will be approved by the Sponsor. Should an amendment be made that requires REC approval, as defined by REC as a substantial amendment, the changes will not be instituted until the amendment has been reviewed and received approval from the REC and R&D departments. A protocol amendment intended to eliminate an apparent immediate hazard to participants may be implemented immediately providing that the REC are notified as soon as possible and an approval is requested. Minor amendments, as defined by REC as nonsubstantial amendment, may be implemented immediately, and the REC will be informed.

The study staff will ensure that participant anonymity is maintained. The participants will be identified only by initials and a participant ID number on the Clinical Record Form and any electronic database. All documents will be stored securely and only accessible by study staff and authorized personnel. The study will comply with the Data Protection Act, which requires data to be anonymized as soon as it is practical to do so.

The study will be performed in accordance with the spirit and letter of the Declaration of Helsinki, the Good Clinical Practice Guidelines, the protocol, and applicable local regulatory requirements and laws.

All study staff must hold evidence of appropriate Good Clinical Practice training or undergo Good Clinical Practice training prior to undertaking any responsibilities on this trial. This training should be updated every 2 years in accordance with trust policy.

### Discontinuation/Withdrawal of Participants

Participants may withdraw at any point in the study during the 6-month follow-up period.

### Definition of End of Study

The end of study is the date of the last data collection from GP record or participant questionnaire 6 months after recruitment.

### Interventions

The participants will have attended a new service, the MISSION clinics, but the care they receive will be no different to standard care according to local and national guidelines.

The difference of the MISSION clinic is (1) the active case finding of patients who are not already known to secondary care but are potentially uncontrolled or who are not known to have COPD and (2) the model of the clinic. Patients seen at the Rapid COPD Assessment Clinic only will undergo more extensive testing and review of their COPD than primary care can provide and will have their comorbidities identified and treatment changes made appropriately.

Patients attending the Severe COPD Assessment Clinic are seen and undergo multidisciplinary review and investigation. The review includes full lung function, inhaler technique, and medical review for all patients and physiotherapy, dietitian, oxygen assessment, CT chest or sinus, psychologist, and smoking cessation as required. Participants will continue to receive their standard treatment.

### Quantitative Data Collection

#### Patients

Notes review will be performed after the MISSION clinics to collect the following data. Where information is not written in medical notes or clinic letters, it will be marked as not assessed. Information will include:

- medical history including COPD history, triggers, allergy history, past medical history, family history, occupation
- lung function
- medication history including COPD and non-COPD medication for comorbidities and related conditions
- exacerbation history including number of steroid courses, hospital admissions, and intensive treatment unit admissions
- health care usage including nonroutine GP attendances, out-of-hour contacts, and ED attendances in 6 months before and after MISSION SAAC or outpatient clinic
- smoking status and if current smoker, whether any smoking cessation advice or referral given
- inhaler technique (if on inhaler) – whether checked, any improvements made, and recommendations for inhaler devices
- comorbidities assessed
- exercise tolerance (using VSAQ plus clinical history)
- questionnaires (PAM, SGRQ, CAT) at baseline and 3 months for all patients
- questionnaires (VSAQ, PAM, CAT, SGRQ, HADS) at baseline and 6 months for patients attending Severe COPD Assessment Clinic
- questionnaires (PAM, CAT, SGRQ) at baseline and 6 months for patients attending Rapid Access COPD Clinics

All participants will be given a self-completion questionnaire, which will be developed in conjunction with Patient Public Involvement advisors.

The areas covered will include the participants’ views about their COPD as well as their views on MISSION using a combination of questions with Likert-scale responses and free text answers. The results will be analyzed for content themes and descriptive statistics such as percentages. The questions may include, for example:

- How confident do you feel managing your COPD?
- How was the education and care from GP/hospital/practice nurse?
- How satisfied were you with the booking process for the clinic / the information that you were given/the team that welcomed you?
- Would you recommend the clinic to family and friends?
- What would you like to change about the clinic?
- Has the clinic improved your knowledge of COPD?

#### Health Care Professionals

All health care professional participants will be given a questionnaire, which will be developed in conjunction with a Patient Public Involvement advisor. The areas covered will include their views on MISSION, problems, barriers, and positive experiences, as well as their experience of COPD care, education, and areas of good practice and areas for improvement using Likert scales and free text answers.

### Qualitative Assessment

#### Patients

All patients will be asked if they wish to have a short one-to-one interview (approximately 30 minutes) to give their views on the clinic. The interview will be held by a member of the research team, and the participant will be invited to say anything they would like to about the clinic. Interviews will be held by telephone within 2 months of the clinic visit.

#### Health Care Professionals

All health care professionals will be asked if they wish to attend a group interview at the end of the clinic to give their views on the clinic. The interview will be facilitated by a member of the research team, and the health care professionals will be invited to say anything they would like to about the clinic they have just taken part in.

### Qualitative Data Analysis

The interviews will be digitally audio recorded and transcribed by a professional transcription company. The data will be entered into a software program to facilitate qualitative analysis (NVivo10). All participants’ names will be removed from the transcripts to retain confidentiality. When writing the results, quotes will be used that represent key themes in the data. However, no quotes will be directly attributed to participants.

The data from open-ended questions will be entered into NVivo10 to facilitate qualitative analysis. All participants’ names will be removed from the data to retain confidentiality. When writing the results, no quotes will be directly attributed to participants.

The results from the questionnaires and interview will be analyzed using a thematic and framework analysis that uses a 5-step approach to analyzing and writing up data [8]. This involves familiarization with the data, identifying themes, indexing the themes onto the data, charting, and mapping the themes. The themes from the patient and health professionals’ questionnaires and interviews can be compared. This will enable key themes to be systematically identified and to map the themes from the patients against those from the health care professionals.

### Patient Public Involvement

The MISSION project was developed by members of the clinical team at Portsmouth after the experience of seeing patients in clinic. MISSION COPD has been developed after a successful MISSION asthma project and feedback from patients who attended.

A patient advisor will review and give feedback on all patient-facing documentation for the study. A patient volunteer will be invited to review the PIS and give advice and feedback on the qualitative questionnaire.

Patient volunteers and patients who have taken part in the study and express interest will be invited to help with dissemination events. The results will be submitted for presentation at national conferences.

All study participants will be given a summary of the study results in an appropriate format.

## Results

The results will be submitted for publication in summer 2017.

## Discussion

### Principal Considerations

COPD affects over 1 million people in the United Kingdom and 1 person dies from COPD every 20 minutes [1]. The cost to people with COPD and the NHS is huge—more than 24 million working days a lost a year and the annual spend on COPD is £810 million [2].

Portsmouth has a higher rate of smoking, which is the commonest cause of COPD, as well as a much higher rate of death caused by smoking than the average for England [4]. Over 900 people were admitted to Portsmouth Hospitals NHS Trust because of COPD in 2013/2014.

The aim of MISSION COPD is to reduce hospital admissions and improve peoples’ quality of life. A project in Southampton where a team looked for people with undiagnosed COPD reduced hospital admissions even though they increased the number of people with a diagnosis of COPD [6].

Our clinics will find patients with severe COPD or with undiagnosed COPD and review them in a one-stop clinic held at their GP surgery—a Rapid Access COPD Clinic. Patients who need more tests will then be seen at a Severe COPD Assessment Clinic at the hospital. Our goal is to see if patients who come to the MISSION clinics have an improvement in their COPD symptoms in the 6 months after the clinic compared to the 6 months before the clinic.

All patients who come to the MISSION COPD clinic will be asked to participate in the research. There are two parts to the research – data analysis and interview. All patients will be invited to take part in both parts, but not everyone will have an interview. Data will be collected from the notes and entered onto a record form anonymously, which will then be analyzed. Interviews will be held over the telephone at a time convenient to the participant. Health care professionals will be invited to take part in a focus group interview at the end of the clinic.

### Strengths and Limitations

This is a small study that will establish the feasibility and successes of the MISSION project. As such, it is not powered for statistical significance. However, its small size allows for greater review of the process through staff and patient experiences allowing the model and project to be changed for future delivery.

### Conclusion

This research is important as it will provide evidence to support the use of the MISSION clinic model for patients with COPD. It will also give us more information on patients with COPD and any medical conditions related to their COPD so that we can adapt our service to meet their needs. The telephone and group interviews will help us understand what we are doing right and what we are doing wrong with COPD care.

## Abbreviations

CAT: COPD Assessment Test
COPD: chronic obstructive pulmonary disease
CT: computerized tomography
ED: emergency department
ESS: Epworth Sleepiness Score
FEV1: forced expiratory volume in 1s
FVC: forced vital capacity
GERD-Q: Gastroesophageal Reflux Disease Questionnaire
GP: general practitioner or general practice
HADS: Hospital Anxiety and Depression Score
MISSION: Modern Innovative SolutionS Improving Outcomes iN
NHS: National Health Service
NHS-IQ: NHS-Improving Quality
PAM: Patient Activation Measure
PIS: Participant Information Sheet
R&D: Research and Development
REC: Research Ethics Committee
SGRQ: St George’s Respiratory Questionnaire
VSAQ: Veterans Specific Activity Questionnaire
